# Treatment preferences among individuals with primary hyperoxaluria type 1 (PH1): a real-world study

**DOI:** 10.1186/s13023-025-03738-9

**Published:** 2025-05-14

**Authors:** David S. Goldfarb, Jing Voon Chen, Rebekah Zincavage, Brad Padilla, Matthew Sussman, Sandra Salem, Frank Modersitzki

**Affiliations:** 1 Division of Nephrology, NYU Langone Health and NYU Grossman School of Medicine, New York, NY USA; 2 Genesis Research Group, Hoboken, NJ USA; 3 Stratevi, Santa Monica, CA USA; 4 Novo Nordisk Inc, Plainsboro, NJ USA

**Keywords:** Primary hyperoxaluria, Real-world study, Patient survey, Treatment preferences

## Abstract

**Background:**

Primary hyperoxaluria type 1 (PH1) is a rare genetic disorder causing excessive oxalate production, damaging kidneys and other organs. Nedosiran, launched in the U.S. for individuals with PH1 (≥ 9 years of age; estimated glomerular filtration rate [eGFR] ≥ 30 mL/min/1.73 m^2^), can be self- or caregiver-administered at home with fixed-dosing for patients ≥ 12 years of age. This real-world study aimed to understand treatment preferences among individuals with PH1, highlighting challenges in administration of current treatments.

**Methods:**

A cross-sectional web-based survey was conducted among U.S.-based adults (aged ≥ 18) diagnosed with PH1. The survey consisted of a 20–25 min questionnaire and was conducted from October to December 2023.

**Results:**

The study participants (*N* = 39) included both male (*N* = 26) and female (*N* = 13) adults with PH1. Participants came from a range of community settings, including urban (46%), rural (39%), and suburban (15%); and were full- or part-time workers (56%) or students (41%). Most participants were on lumasiran therapy (95%) for an average of 1 year (range: 0.3–1.8 years). The survey revealed that the commonly reported factors important for treatment selection among participants living with PH1 were frequency of administration, treatment administrator, time required for treatment, and place of administration. The ability to self-administer was ranked as the top choice by most participants. Over half (56%) found quarterly injections easy or very easy. Similarly, 56–59% found home administration, whether self- or healthcare provider (HCP)-administered, easy or very easy. Nearly half (46%) considered injections at medical facilities challenging or very challenging. The majority indicated traveling > 15 min for injections would be burdensome (57%) and arranging appointments problematic (54%). When comparing administration methods, 72% preferred self-injection over HCP-administered injections. Regarding treatment regimens, 57% found it easy or very easy to receive monthly injections initially, before switching to quarterly. Additionally, 64% preferred a medication dosage that is not weight-based. While participants expressed a preference for less frequent treatments, 67% preferred self-injection at home over medical facility injections, and 67% preferred monthly injections at home over quarterly injections at a medical facility.

**Conclusions:**

This study shows that patients with PH1 value treatments that are convenient and fit their lifestyle.

**Supplementary Information:**

The online version contains supplementary material available at 10.1186/s13023-025-03738-9.

## Background

Primary hyperoxaluria (PH) is a rare autosomal recessive disorder leading to excessively elevated levels of endogenous oxalate in the plasma and urine, leading to kidney and other organ damage [1, 2]. Primary hyperoxaluria type 1 (PH1), the most common and severe form, is estimated to have a genetic prevalence of 1 in 151,887 births [3]. PH1 accounts for approximately 80% of clinically diagnosed cases, while types 2 (PH2) and 3 (PH3) each account for about 10% of cases each [3]. If left untreated, the disease can cause kidney failure (e.g., end-stage renal disease [ESRD]), organ damage and premature death [4, 5].

Patients with PH1 have limited treatment options and significant healthcare resource utilization, costs, and poor quality of life [6]. Treatment of PH1 involves the reduction of calcium oxalate (CaOx) crystal formation in the kidneys through hyperhydration, dietary adjustments, and potassium citrate and pyridoxine (vitamin B6) intake [7]. The first RNAi therapy approved for PH1, lumasiran, has also been used to lower urine and plasma oxalate (Uox and Pox) levels and is administered subcutaneously by a healthcare provider (HCP) quarterly after three months of loading doses [8–10]. Nedosiran, a novel RNAi therapy, inhibits the production of hepatic lactate dehydrogenase (LDH) enzyme in the glyoxylate metabolism pathway to prevent the overproduction of oxalate [11–13]. The hepatic LDH enzyme inhibition occurs only in the liver due to the incorporation of N-acetylgalactosamine (GalNAc)-targeting ligands present in nedosiran, which bind specifically to the asialoglycoprotein receptors (ASGPRs) predominantly expressed on hepatic cell surfaces [14]. The PHYOX2 (NCT03847909) and PHYOX3 (NCT04042402) trials observed that over 80% of patients with PH1 receiving nedosiran reached normal or near-normal Uox excretions at end of study follow up, demonstrating that nedosiran represents a treatment option for these patients [15, 16]. Furthermore, as a monthly self-administered or caregiver-administered subcutaneous (s.c.) fixed-dose injection (pre-filled syringe for patients 9 years and older, weighing ≥ 50 kg) at home with no loading dose required, nedosiran has the potential to alleviate patient burden and perhaps improve potential adherence to treatment. The primary objectives of this study were to: (i) explore the preferences of individuals with PH1 regarding five key treatment attributes: frequency and place of administration, subject who administers, complexity of regimen, and time requirement; and (ii) characterize the specific challenges associated with PH1 treatment administration. By highlighting patient preferences in the real world, this study has the potential to raise awareness to the treatment experience of individuals with PH1 and understand drivers and preferences of treatment selection from a patient perspective.

## Methods

### Study design and participants

**Fig. 1:**
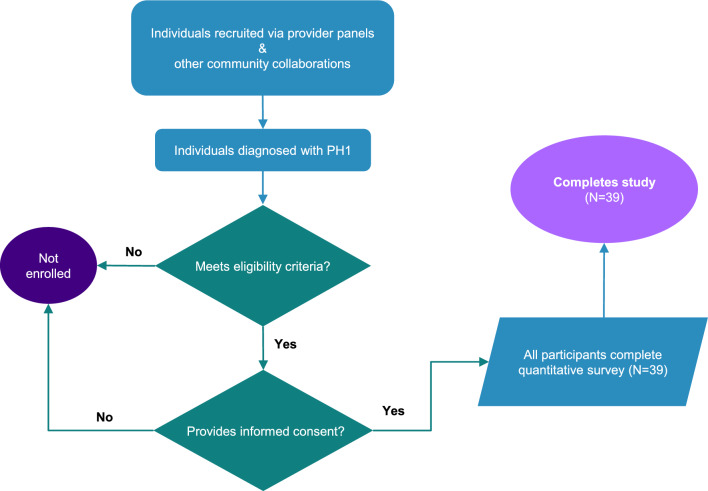
Study design and participants. Participant overview and study flow. PH1, primary hyperoxaluria type 1

This survey was a virtual, cross-sectional study that enrolled 39 adults (aged ≥ 18) who had a diagnosis of PH1. Participants meeting the study eligibility criteria were asked to electronically complete an informed consent form (ICF) and questionnaire via their smartphones or computers (Fig. 1). The questionnaire was designed to take approximately 20–25 min to complete. The study was executed utilizing the inVibe Labs (inVibe) technology platform, which enables data collection via web-enabled questionnaires. After participants completed the online questionnaire, they were provided an honorarium for their participation. The study was conducted in accordance with the European Pharmaceutical Market Research Association (EphMRA) Code of Conduct and the provisions of the Declaration of Helsinki. Before use, the ICF was reviewed by the responsible party and approved by the WCG Institutional Review Board (IRB). Participants were allowed sufficient time to consider participation in the study. By signing the ICF, participants agreed to participate in the study unless they withdrew voluntarily or were terminated from the study for any reason.

### Study population

Key inclusion criteria were ≥ 18 years of age (at the time of signing ICF) and a confirmed PH1 diagnosis. Furthermore, all participants had to complete the study ICF (before any study-related activities) and be living in the United States (U.S.). Key exclusion criteria included prior or planned kidney or hepatic transplantation or dialysis during the study, currently untreated or unmanaged PH1, unwillingness or language barriers (participants were required to understand English) precluding adequate understanding or cooperation. Current Novo Nordisk employees, as well as their immediate family members, were not included in the survey. Individuals could only participate in the survey once and were required to have a mobile device that could send and receive text messages or have access to a computer.

### Data collection

The recruitment was conducted between October 6 th, 2023 and December 21 st, 2023 and the survey was executed utilizing an online technology platform owned by inVibe. Individuals were recruited through provider panels and other community collaborations. Data were collected via a web-enabled screener and questionnaire. Any participant who met the inclusion criteria and did not meet the exclusion criteria as determined by the screener questions, and provided informed consent was enrolled in the study and completed the questionnaire one time via a computer, tablet, and/or mobile device connected to the internet. The main areas included in the questionnaire were: (1) demographics, including questions about employment status, education level, relationship status, and type of community; (2) diagnosis and treatment history, including questions about the age of diagnosis and treatments taken over the last three months to manage PH1 symptoms; (3) treatment preferences, including questions about the most important factors when choosing therapies, ease of use, and preferences for different treatment characteristics.

### Data analysis

Descriptive statistics were used to describe the study population, including participant and treatment characteristics, and the primary outcome measures. Summary tables (descriptive statistics and/or frequency tables) were provided for all variables in the screener and questionnaire as appropriate. Continuous variables, such as age, were summarized using the N (number of responses to the question), mean, standard deviation, minimum, median, and maximum. For categorical variables, such as gender, the number of respondents (N), frequency, and percentage were calculated. The evaluable population included all eligible participants who completed the questionnaire. Completing the questionnaire means reaching the end of the questionnaire and responding to the questions in good faith (i.e., the data is deemed to be of high quality and not suspected to be from a fraudulent respondent). Formal significance testing was not needed as this was an exploratory study, and there were no pre-specified hypotheses.

## Results

### Participant characteristics

**Fig. 2:**
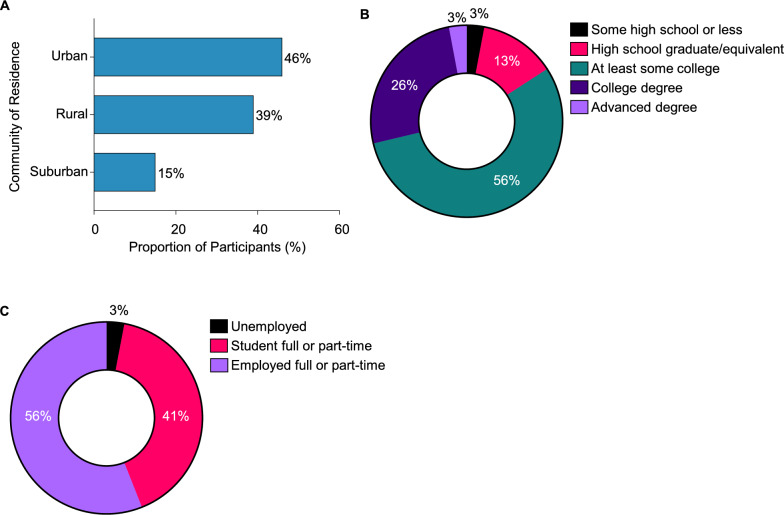
Participant characteristics. **A**. Community of residence. **B**. Highest level of education. **C**. Current employment status

The study included 39 participants (mean age: 28.5 years; range: 19–51 years) with diagnosed PH1. The cohort comprised both male (*N* = 26) and female (*N* = 13) participants, with an average age of PH1 diagnosis at 16 years (Table 1). The participants represented diverse community settings, including urban (46%), rural (39%), and suburban (15%) (Fig. 2A). Most of the participants (84%) had at least some college education or higher (Fig. 2B). A significant portion of the participants were full- or part-time workers (56%) or students (41%) (Fig. 2C).

**Table 1:** Participant characteristics

Age (in years)	(*N* = 39)
Mean (SD)	28.5 (9.4)
Median (Range)	23.0 (19–51)
Gender (n, %)	(*N* = 39)
Male	26 (66.7)
Female	13 (33.3)
Age at PH1 diagnosis (n, %)	(*N* = 39)
Mean (SD)	16.2 (13.5)
Median (Range)	16.3 (1–50)

*SD* standard deviation, *PH1* primary hyperoxaluria type 1

### Treatment history and preferences

**Fig. 3:**
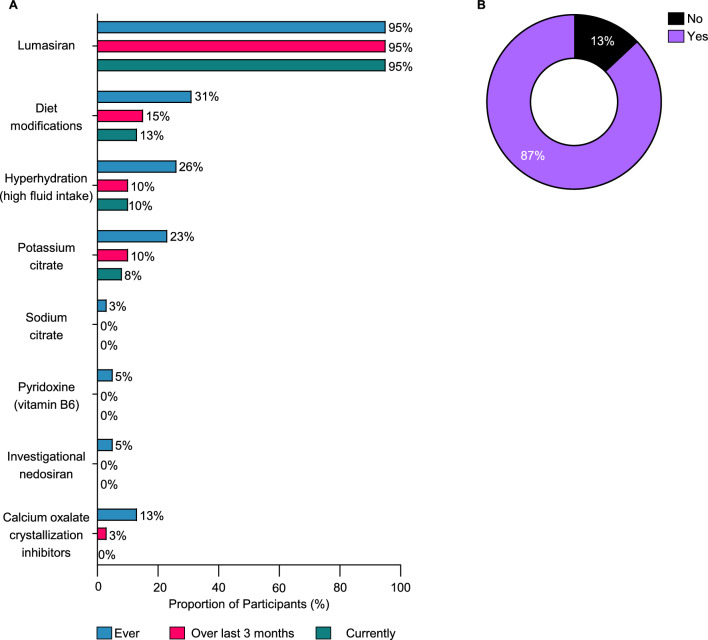
Treatment history and preferences. **A**. PH1 treatments used. **B**. Traveling to receive PH1 treatment. Abbreviations: PH1, primary hyperoxaluria type 1

**Fig. 4:**
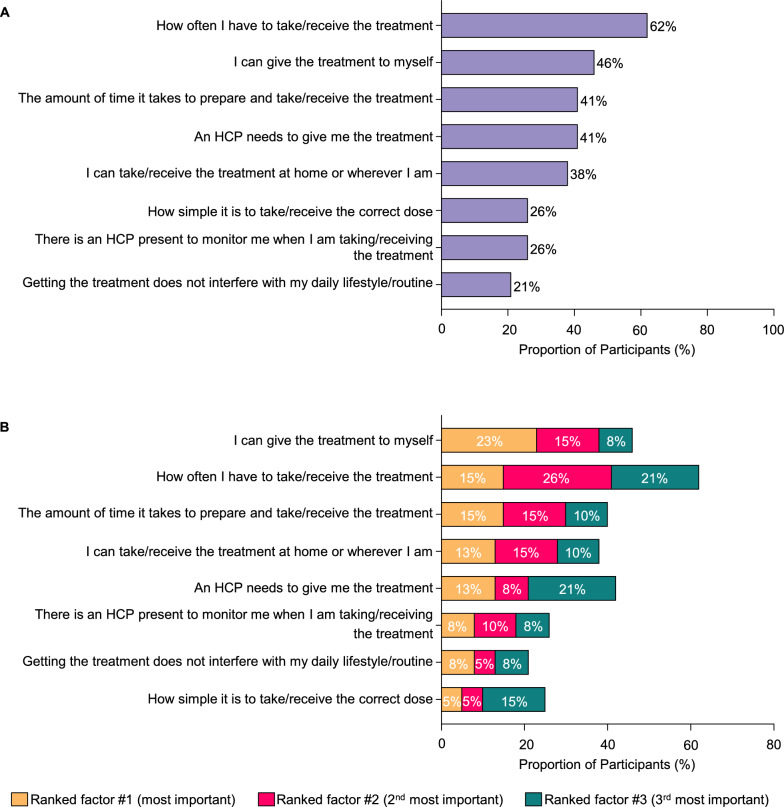
Factors important for treatment selection. **A**. Most important factors when choosing possible therapies for PH1. **B**. Ranking of factors related to the way patients take/receive a therapy. HCP, healthcare provider; PH1, primary hyperoxaluria type 1

Lumasiran therapy was the predominant treatment among study participants (95%), with most being on treatment for an average duration of one year (range: 0.3–1.8 years) (Fig. 3A). A smaller percentage of participants reported the previous use of supplementary approaches, including dietary modifications (31%), hyperhydration (26%), and potassium citrate (23%) (Fig. 3A). Despite its prevalence, lumasiran treatment often required considerable travel and time investment for the recipients. Most participants (87%) reported taking at least one trip to a medical facility exclusively for PH1 treatment, with a mean (standard deviation [SD]) of 6.4 (2.3) trips annually (Fig. 3B and Table 2). Each visit required a substantial time commitment, with a mean (SD) of 4.1 (3.3) hours (range: 1–14 h) (Table 2). The hours spent per visit included time spent scheduling the appointment, traveling to the appointment, waiting to be seen, and traveling home from the appointment. For more than half of the participants (62%), the frequency of treatment administration emerged as the most important factor when choosing possible therapies for PH1, followed by a strong preference for self-administration (46%). The time required for treatment (41%), the convenience of location (38%), and the complexity of the regimen (26%) were also important considerations (Fig. 4A). When participants were asked to rank the factors important when selecting PH1 treatments as “most important”, “2nd most important”, and “3rd most important”, the ability to self-administer treatment was frequently ranked as the most important factor (23%), underscoring the value participants placed on autonomy and ease of treatment. The frequency of treatment was the most common among the top three factors (15, 26, and 21%), highlighting its importance in the daily lives of participants (Fig. 4B). Altogether, the commonly reported attributes important for treatment selection among participants with PH1 were frequency of administration, treatment administrator, time required for treatment, and place of administration.

**Table 2:** Travel experience for treatment

Average number of trips per year made exclusively to receive treatment	(*n* = 34)
Mean (SD)	6.4 (2.3)
Median (Range)	6 (3–12)

Average number of hours spent per visit*	(*n* = 34)
Mean (SD)	4.1 (3.3)
Median (Range)	3.0 (1–14)

*SD* standard deviation

^*^As part of the hours spent per visit, participants were asked to “please consider time spent scheduling the appointment, traveling to the appointment, waiting to be seen, and traveling home from the appointment”

### Domains of treatment attributes evaluated

**Fig. 5:**
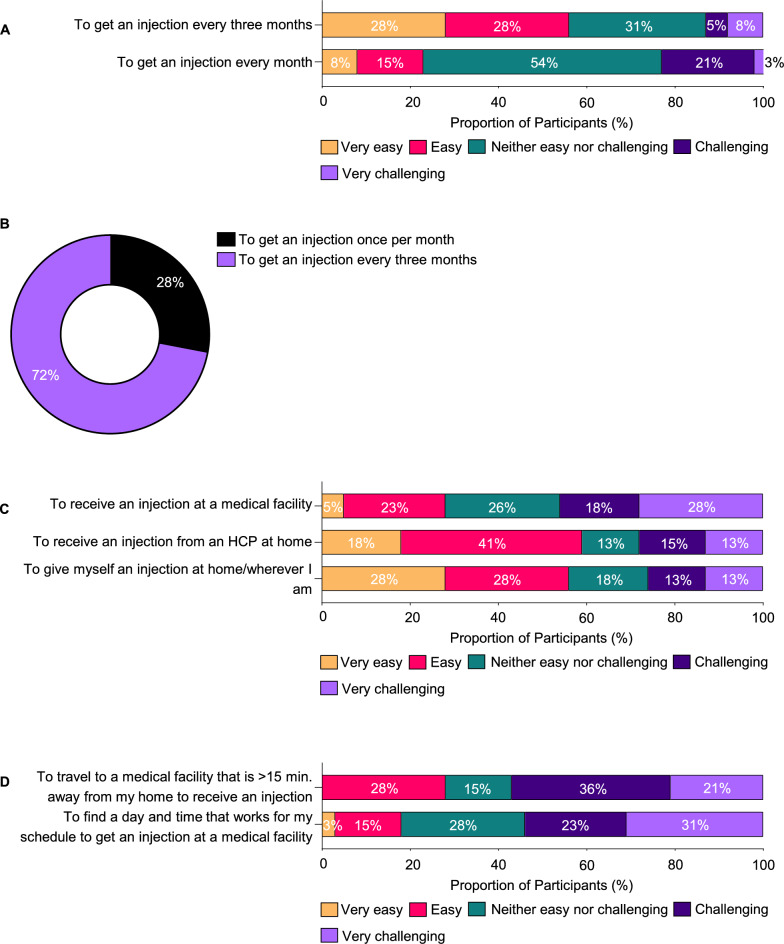
Preferences as single attributes: frequency, location, time and travel. **A**. Treatment ease of use in terms of frequency of injection. **B**. Treatment preference in terms of frequency of injection. **C**. Treatment ease of use in terms of location of injection. **D**. Treatment ease of use in terms of travel and time requirements. HCP, healthcare provider

There was a preference for less frequent injection schedules among participants, with 56% reporting that receiving an injection quarterly was manageable (either “very easy” or “easy”). In contrast, less than 25% found monthly injections to be convenient (Fig. 5A). A significant majority, nearly 75%, favoured quarterly over monthly injections, highlighting a preference for less frequent treatments (Fig. 5B). The challenge of receiving injections at a medical facility was noted by 46% of participants, whereas 56% felt self-administration at home would be easier, and 59% were comfortable with receiving injections at home from an HCP (Fig. 5C). These data suggest that treatment at home is perceived as less challenging, with more than half (57%) of the participants finding it difficult to travel more than 15 min for an injection and 54% considering the scheduling of appointments to be burdensome (Fig. 5D).

**Fig. 6:**
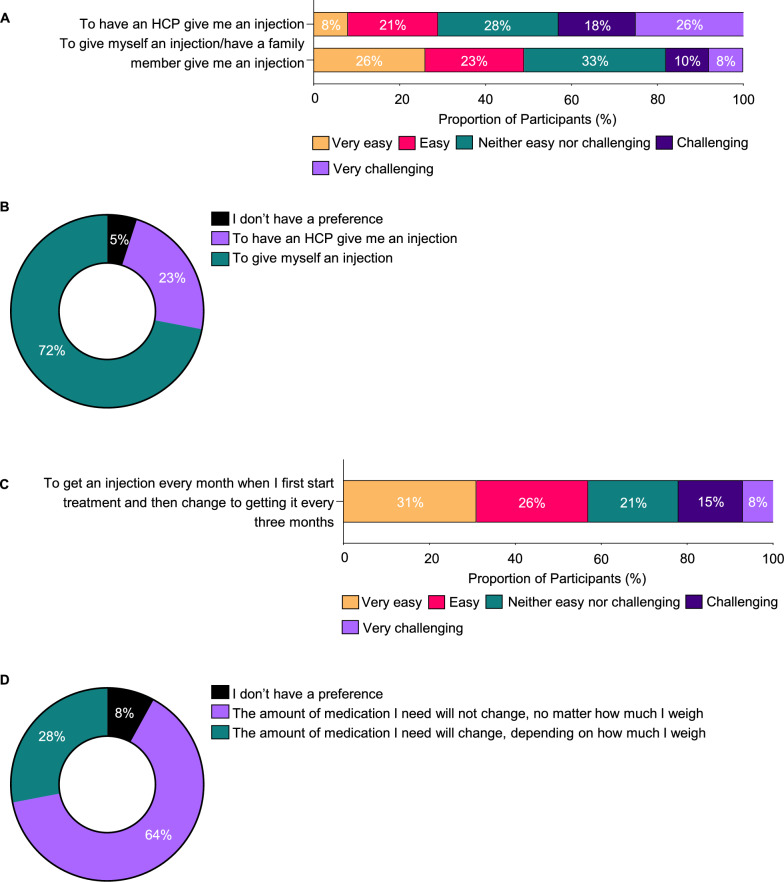
Preferences as single attributes: Treatment Administrator and Complexity. **A**. Treatment ease of use in terms of who administers injection. **B**. Treatment preference in terms of who administers injection. **C**. Treatment ease of use in terms of regimen complexity. **D**. Treatment preference in terms of regimen complexity. PH1, primary hyperoxaluria type 1; HCP, healthcare provider

As previously mentioned, self-administration emerged as a crucial factor in treatment selection, with 46% ranking it among their top three preferences. In line with this, several participants (44%) found it challenging to have HCPs administer their treatments, indicating a preference for more autonomy in managing their treatments (Fig. 6A). Nearly half of the participants, 49%, feel confident in self-administering injections, while only 18% find it challenging (Fig. 6A). Between self-injection and HCP-administered injections, 72% indicated preference for self-injection (Fig. 6B). Additionally, 57% of participants were open to monthly injections initially, transitioning to quarterly, and 64% preferred a medication dosage that does not change with weight, indicating a preference for simpler treatment regimens (Fig. 6C and D). Overall, participants expressed a strong preference for less frequent and less complex PH1 treatment regimens that can be self-administered at home.

**Fig. 7:**
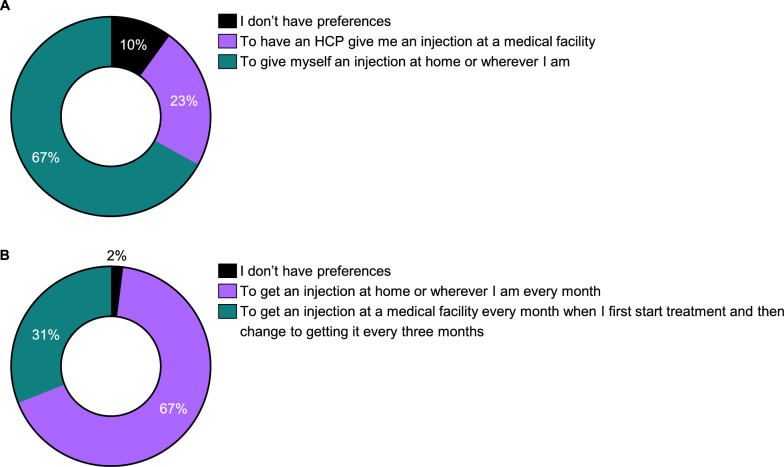
Preferences as combination of attributes: location and frequency. **A**. Treatment preference in terms of location of administration. **B**. Treatment preference in terms of frequency of treatment. HCP, healthcare provider

A substantial majority, 67%, expressed a preference for self-injection at home, compared to the 23% who preferred injections at a medical facility (Fig. 7A). This preference for home-based treatment was further emphasized by the fact that most participants (67%) would opt for monthly injections at home over quarterly injections at a medical facility (Fig. 7B), despite 72% expressing a preference for less frequent treatments (Fig. 5B). This suggests that the convenience of home administration outweighs the desire for less frequent treatment schedules, highlighting the importance of participant convenience and the willingness to manage more frequent treatments if they can be administered in the comfort of their own home.

## Discussion

This survey was a virtual, cross-sectional study that enrolled 39 adults aged 18 or older, who had been diagnosed with PH1. The study aimed to highlight patient preferences in the real world, potentially generating critical evidence that can help raise awareness of the treatment experiences of individuals with PH1.

Most of the study’s participants resided in urban or rural areas, with nearly all either being employed or pursuing education. This demographic data highlights the need for treatment regimens that accommodate busy and mobile lifestyles.

Lumasiran emerged as the most common treatment among participants, yet it often necessitates considerable travel and time, which may not be ideal for this active population. It is important to note that the high prevalence of lumasiran usage among participants was due to nedosiran not being commercially available at the time of this research and not due to preference over nedosiran.

The data indicated a strong preference for self-administration, with participants finding it less challenging and more convenient to receive treatment at home rather than traveling to medical facilities. This preference was further reinforced by the perceived burdens of traveling and scheduling treatments, which are significant factors in the overall management of the condition.

Participants demonstrated a consistent preference for less frequent treatments, which would typically suggest a quarterly injection schedule. However, when considering the location of administration, the convenience of home-based treatments leads to a willingness to accept more frequent injections. This finding is notable, as it suggests that the convenience of self-administration at home may be a more significant factor than the frequency of treatment itself.

Nedosiran has been recently launched in the U.S. for patients living with PH1 (≥ 9 years of age; eGFR ≥ 30 mL/min/1.73 m^2^), as a monthly s.c. injection which can be self- or caregiver-administered at home with the option of fixed-dosing for patients [13]. Based on this, nedosiran has the potential to alleviate patient burden and perhaps improve potential adherence to treatment. Previous studies on chronic conditions have shown that poor adherence to medication can result in suboptimal treatment outcomes, supporting disease progression, and reducing overall quality of life [17]. Adherence to treatment regimens is critical in managing PH, as inconsistent treatment can potentially lead to chronic kidney disease (CKD) progression and other organ damage [4, 5, 18]. A retrospective cohort study highlighted how hyperoxaluric patients compliant in reducing consumption of oxalate-containing foods and increasing fluid intake were significantly less likely to require interventions for urinary stones within 24 months compared to non-compliant patients [19]. Although the cohort consisted of non-primary hyperoxaluria patients, these findings suggest the importance of treatment compliance for PH. The implications of our study’s findings are substantial for participant adherence and satisfaction. Treatments that are less complex, less frequent, and can be self-administered at home are likely to result in better adherence to treatment regimens and higher participant satisfaction. This, in turn, has the potential to enhance the overall management of the condition and perhaps lead to better health outcomes for patients.

The results of our study come from a sample size of 39 participants, which may limit the generalizability of the findings to the broader PH1 patient population. However, PH1 is a rare disease, and real-world studies often have smaller sample sizes due to the limited number of patients. Additionally, the study examined individual treatment attributes in a blinded format, meaning participants were not directly presented with complete treatment profiles. As most participants were familiar with lumasiran and its features, while only one participant had experience with investigational nedosiran, this familiarity may have introduced bias into the study findings. However, the directionality and magnitude of this potential bias remain unclear. Despite these limitations, the clinicians included among the authors believe that these findings will inform future clinical research and the development of treatment regimens that align with patient preferences, ultimately improving patient outcomes and satisfaction.

## Conclusion

In conclusion, this study provides valuable insights into patient preferences and treatment experiences in the real world. These results highlight the importance of considering participant convenience and lifestyle when developing treatment regimens for PH1, suggesting that treatments aligning with these preferences are more likely to be successful in real-world settings.

## Supplementary Information

Additional file 1

## Data Availability

The data that support the findings of this study are not openly available due to reasons of sensitivity. The data used and/or analysed during the current study are, however, available from the corresponding author and with permission of the participants upon reasonable request.

## Abbreviations

ASGPRs: Asialoglycoprotein receptors
CaOx: Calcium oxalate
CKD: Chronic kidney disease
eGFR: Estimated glomerular filtration rate
EphMRA: European pharmaceutical market research association
ESRD: End-stage renal disease
GalNAc: N-Acetylgalactosamine
HCP: Healthcare provider
ICF: Informed consent form
IRB: Institutional review board
LDH: Lactate dehydrogenase
PH: Primary hyperoxaluria
PH1: Primary hyperoxaluria type 1
PH2: Primary hyperoxaluria type 2
PH3: Primary hyperoxaluria type 3
Pox: Plasma oxalate
RNAi: RNA interference
s.c.: Subcutaneous
SD: Standard deviation
Uox: Urine oxalate

## References

[1] Cochat P, Rumsby G. Primary hyperoxaluria. N Engl J Med. 2013;369(7):649–58. 23944302 10.1056/NEJMra1301564

[2] Hoppe B. An update on primary hyperoxaluria. Nat Rev Nephrol. 2012;8(8):467–75. 22688746 10.1038/nrneph.2012.113

[3] Hopp K, Cogal AG, Bergstralh EJ, Seide BM, Olson JB, Meek AM, et al. Phenotype-genotype correlations and estimated carrier frequencies of primary hyperoxaluria. J Am Soc Nephrol. 2015;26(10):2559–70. 25644115 10.1681/ASN.2014070698 PMC4587693

[4] Edvardsson VO, Goldfarb DS, Lieske JC, Beara-Lasic L, Anglani F, Milliner DS, et al. Hereditary causes of kidney stones and chronic kidney disease. Pediatr Nephrol. 2013;28(10):1923–42. 23334384 10.1007/s00467-012-2329-z PMC4138059

[5] Mandrile G, van Woerden CS, Berchialla P, Beck BB, Acquaviva Bourdain C, Hulton SA, et al. Data from a large European study indicate that the outcome of primary hyperoxaluria type 1 correlates with the AGXT mutation type. Kidney Int. 2014;86(6):1197–204. 24988064 10.1038/ki.2014.222

[6] Mucha L, Hoppe B, Silber A, Wang Z, Miyasato G, Skaar JR, et al. Clinical and economic impact of primary hyperoxaluria: a retrospective claims analysis. J Manag Care Spec Pharm. 2022;28(3):316–23. 35199581 10.18553/jmcp.2022.28.3.316 PMC10373026

[7] Groothoff JW, Metry E, Deesker L, Garrelfs S, Acquaviva C, Almardini R, et al. Clinical practice recommendations for primary hyperoxaluria: an expert consensus statement from ERKNet and OxalEurope. Nat Rev Nephrol. 2023;19(3):194–211. 36604599 10.1038/s41581-022-00661-1

[8] Alnylam Pharmaceuticals Inc. Oxlumo 2022.

[9] European Medicines Agency. Oxlumo2020.

[10] Food and Drug Administration, CDER. Oxlumo2020 30-Mar. Available from: https://www.accessdata.fda.gov/drugsatfda_docs/nda/2020/214103Orig1s000TOC.cfm.

[11] Food and Drug Administration CfDEaR. Rivfloza (Nedosiran), NDA Integrated Review. September 2023.

[12] Novo Nordisk A/S. Rivfloza (nedosiran), US Prescribing Information (PI)2023.

[13] Syed YY. Nedosiran: first approval. Drugs. 2023;83(18):1729–33. 38060091 10.1007/s40265-023-01976-4 PMC10803381

[14] Liu A, Zhao J, Shah M, Migliorati JM, Tawfik SM, Bahal R, et al. Nedosiran, a candidate siRNA drug for the treatment of primary hyperoxaluria: design, development, and clinical studies. ACS Pharmacol Transl Sci. 2022;5(11):1007–16. 36407951 10.1021/acsptsci.2c00110 PMC9667536

[15] Baum MA, Langman C, Cochat P, Lieske JC, Moochhala SH, Hamamoto S, et al. PHYOX2: a pivotal randomized study of nedosiran in primary hyperoxaluria type 1 or 2. Kidney Int. 2023;103(1):207–17. 36007597 10.1016/j.kint.2022.07.025

[16] Groothoff J, Sellier-Leclerc AL, Deesker L, Bacchetta J, Schalk G, Tönshoff B, et al. Nedosiran safety and efficacy in PH1: interim analysis of PHYOX3. Kidney Int Rep. 2024;9(5):1387–96. 38707801 10.1016/j.ekir.2024.02.1439 PMC11068990

[17] Burnier M. The role of adherence in patients with chronic diseases. Eur J Intern Med. 2024;119:1–5. 37479633 10.1016/j.ejim.2023.07.008

[18] Cedillo-Couvert EA, Ricardo AC, Chen J, Cohan J, Fischer MJ, Krousel-Wood M, et al. Self-reported medication adherence and CKD progression. Kidney Int Rep. 2018;3(3):645–51. 29854972 10.1016/j.ekir.2018.01.007 PMC5976857

[19] Hennessey DB, Kinnear N, Rice G, Curry D, Woolsey S, Duggan B. Compliance in patients with dietary hyperoxaluria: a cohort study and systematic review. Asian J Urol. 2019;6(2):200–7. 31061807 10.1016/j.ajur.2018.03.002 PMC6488745

