# Supplementary material for: Treatment preferences among individuals with primary hyperoxaluria type 1 (PH1): a real-world study
Source: Orphanet J Rare Dis. 2025 May 14;20:228. doi: 10.1186/s13023-025-03738-9 (PMC12080053; doi:10.1186/s13023-025-03738-9)
Supplement: Supplementary file 1 — Additional file 1 [file 13023_2025_3738_MOESM1_ESM.pptx]

## Slide 1
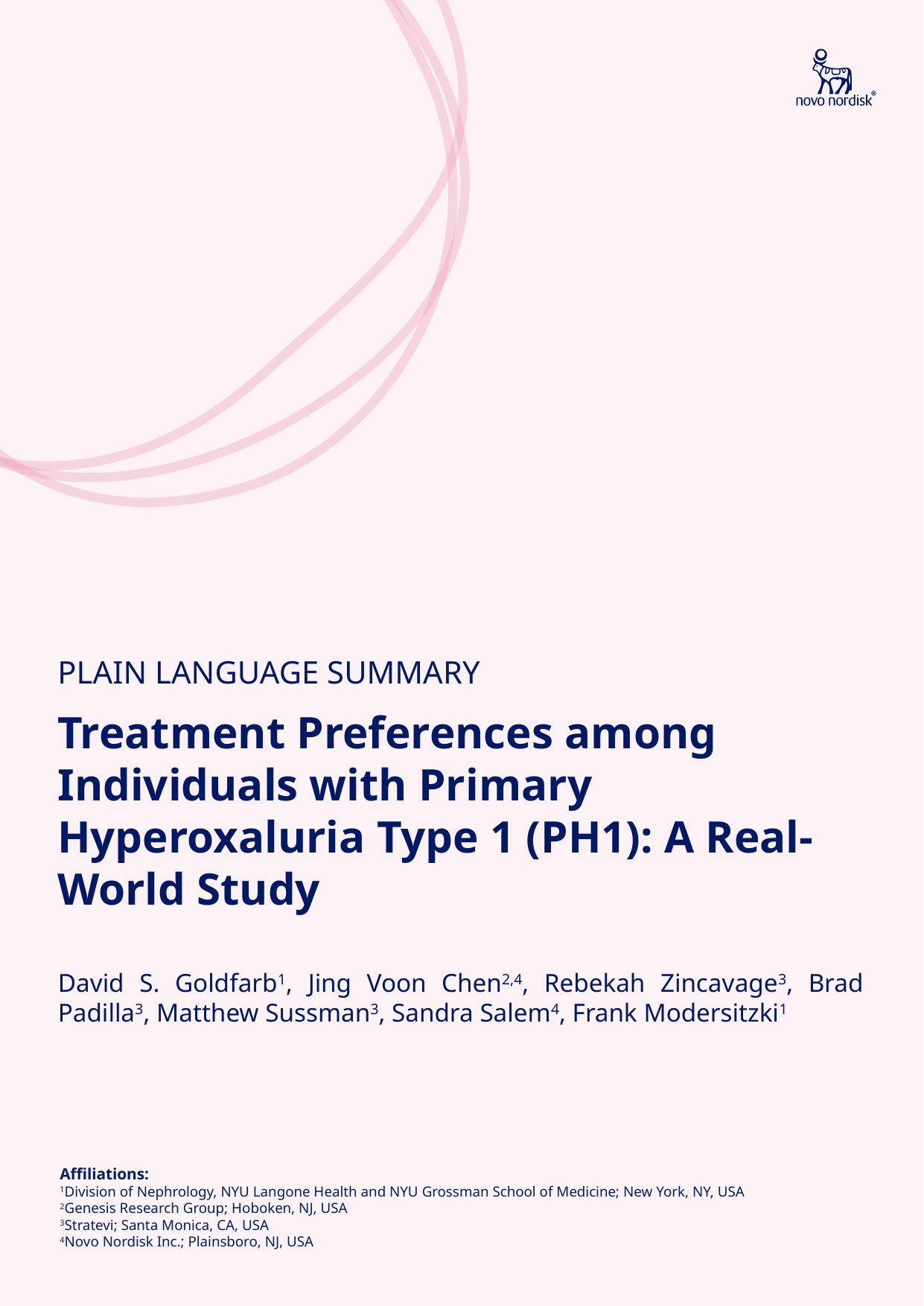

PLAIN LANGUAGE SUMMARY
# Treatment Preferences among Individuals with Primary Hyperoxaluria Type 1 (PH1): A Real-World Study
David S. Goldfarb1, Jing Voon Chen2,4, Rebekah Zincavage3, Brad Padilla3, Matthew Sussman3, Sandra Salem4, Frank Modersitzki1
Affiliations:
1Division of Nephrology, NYU Langone Health and NYU Grossman School of Medicine; New York, NY, USA
2Genesis Research Group; Hoboken, NJ, USA
3Stratevi; Santa Monica, CA, USA
4Novo Nordisk Inc.; Plainsboro, NJ, USA
.
.

## Slide 2
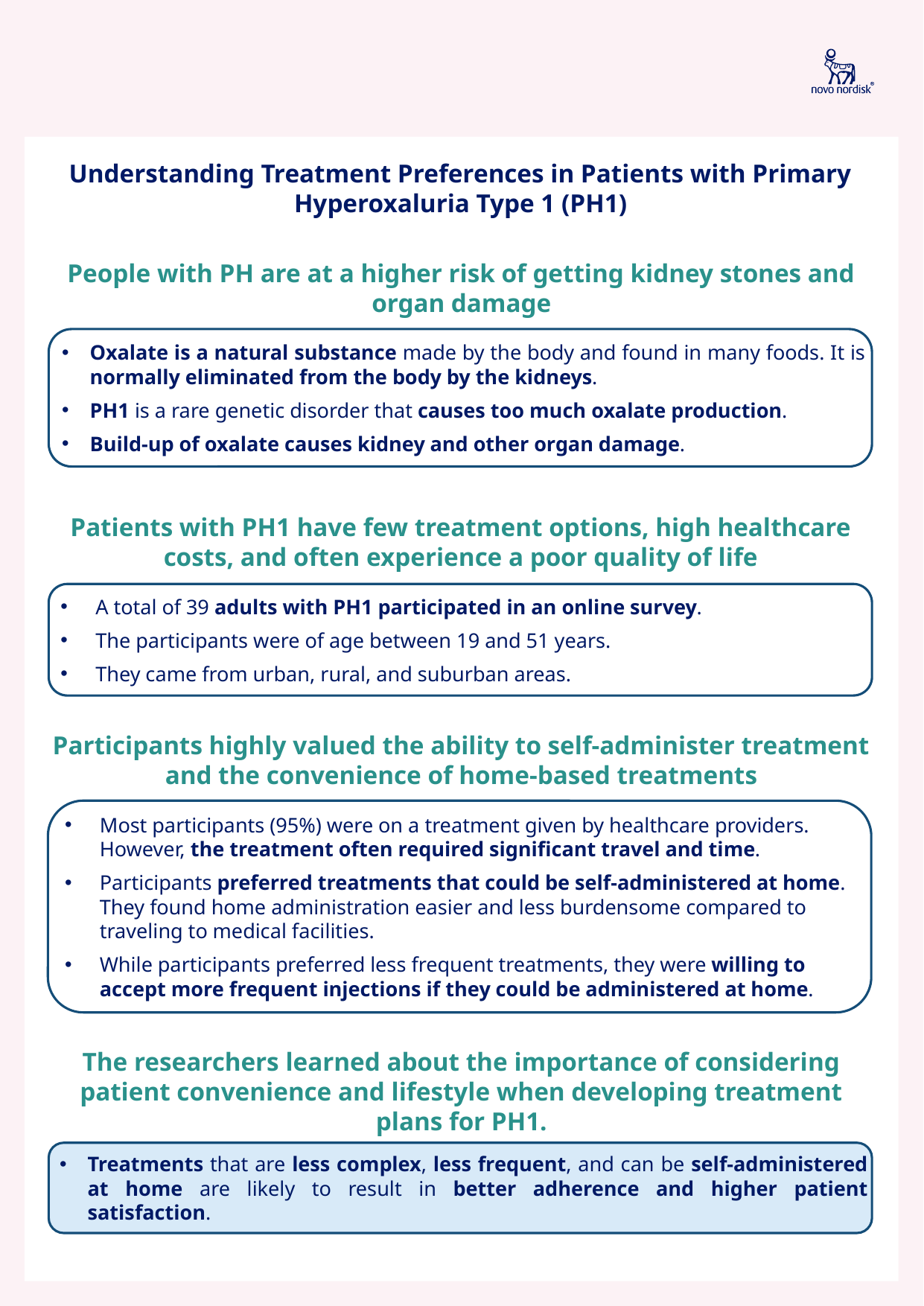

# Understanding Treatment Preferences in Patients with Primary Hyperoxaluria Type 1 (PH1)
People with PH are at a higher risk of getting kidney stones and organ damage
Oxalate is a natural substance made by the body and found in many foods. It is normally eliminated from the body by the kidneys.
PH1 is a rare genetic disorder that causes too much oxalate production.
Build-up of oxalate causes kidney and other organ damage.
Patients with PH1 have few treatment options, high healthcare costs, and often experience a poor quality of life
A total of 39 adults with PH1 participated in an online survey.
The participants were of age between 19 and 51 years.
They came from urban, rural, and suburban areas.
Participants highly valued the ability to self-administer treatment and the convenience of home-based treatments
Most participants (95%) were on a treatment given by healthcare providers. However, the treatment often required significant travel and time.
Participants preferred treatments that could be self-administered at home. They found home administration easier and less burdensome compared to traveling to medical facilities.
While participants preferred less frequent treatments, they were willing to accept more frequent injections if they could be administered at home.
The researchers learned about the importance of considering patient convenience and lifestyle when developing treatment plans for PH1.
Treatments that are less complex, less frequent, and can be self-administered at home are likely to result in better adherence and higher patient satisfaction.
